# Dynamic Hubs Show Competitive and Static Hubs Non-Competitive Regulation of Their Interaction Partners

**DOI:** 10.1371/journal.pone.0048209

**Published:** 2012-10-31

**Authors:** Apurv Goel, Marc R. Wilkins

**Affiliations:** Systems Biology Initiative, School of Biotechnology and Biomolecular Sciences, University of New South Wales, Sydney, Australia; Texas A&M University, United States of America

## Abstract

Date hub proteins have 1 or 2 interaction interfaces but many interaction partners. This raises the question of whether all partner proteins compete for the interaction interface of the hub or if the cell carefully regulates aspects of this process? Here, we have used real-time rendering of protein interaction networks to analyse the interactions of all the 1 or 2 interface hubs of *Saccharomyces cerevisiae* during the cell cycle. By integrating previously determined structural and gene expression data, and visually hiding the nodes (proteins) and their edges (interactions) during their troughs of expression, we predict when interactions of hubs and their partners are likely to exist. This revealed that 20 out of all 36 one- or two- interface hubs in the yeast interactome fell within two main groups. The first was dynamic hubs with static partners, which can be considered as ‘competitive hubs’. Their interaction partners will compete for the interaction interface of the hub and the success of any interaction will be dictated by the kinetics of interaction (abundance and affinity) and subcellular localisation. The second was static hubs with dynamic partners, which we term ‘non-competitive hubs’. Regulatory mechanisms are finely tuned to lessen the presence and/or effects of competition between the interaction partners of the hub. It is possible that these regulatory processes may also be used by the cell for the regulation of other, non-cell cycle processes.

## Introduction

The construction and analysis of protein-protein interaction networks has revealed that they are heterogeneous and have many interesting features. One example of a network feature is the hub protein; these are highly connected proteins which, typically, in yeast have 5 or more interactions [1]. There is much discussion about the biological importance of hubs. Some groups have suggested an association between hubs and essentiality (the ‘centrality-lethality hypothesis’) [2]–[4] but other studies have suggested that the evolutionary conservation of hubs [5] and their relationship with pleiotropy [6] are of greater importance. Hubs are proposed to be of two main types: ‘date’ hubs, which interact with their partners at different times and at different locations, and ‘party’ hubs which interact with most of their partners simultaneously as part of large stable complexes [1].

While the absolute distinction between date and party hubs remains under debate [7], [8], structural analysis of hub proteins provides a means of assessing how a protein is likely to interact with its partners and how many interactions it can participate in at once [9]. Kim *et al.* (2006) [10] structurally analysed all hub proteins in yeast to determine the number of interaction interfaces per protein. Hubs could then be classified as ‘singlish’ (1 or 2 interaction interfaces) or ‘multi interface’ (3 or more interaction interfaces). With only 1 or 2 interaction interfaces, singlish hubs must essentially interact with their partners one at a time, in a mutually exclusive manner. Multi interface proteins, by contrast, are likely to be part of large and stable complexes, allowing the interaction with several proteins at once. These observations are supported by differences in the intrinsic disorder of hub types, whereby date hubs show high intrinsic disorder, suggesting a capacity for transient binding and flexible interfaces [11]–[14].

The co-analysis of protein interaction networks and time-series expression data is a powerful means to investigate the dynamics of interaction networks [15]–[17]. Using time-series gene expression data from the cell cycle, de Lichtenberg *et al.* examined peak expression time for proteins in the context of multiprotein complexes [15]. From this, the authors described a novel means by which the cell regulates the construction of protein complexes. Called ‘just in time assembly’, the majority of proteins in a complex are constitutively expressed but one protein (essential to the function of the complex) is dynamically expressed when required. This activates the function of the whole complex and is an efficient means of doing so as it obviates the need for the regulation and expression of all genes for all members of a protein complex. It is of particular importance in situations such as the cell cycle, where the cell needs to activate certain complexes at a precise time.

The discovery of just in time assembly for the regulation of multiprotein complexes, including party hub proteins, raises the question of how the cell regulates the expression and thus interactions of date hub proteins and their many partners. Do the partners compete with each other to bind to a hubs interaction interface or are there regulatory mechanisms that dictate strict expression of either the hub and/or partner protein? A previous study analysed peak expression times of hubs and their interaction partners within the cell cycle, revealing that most hubs show dynamic interactions with their partners [18]. However, the number and type of interaction interfaces of each hub, and regulatory processes specifically associated with one- or two-interface hubs, were not considered. Using 4-D real time rendering [16], a technique to dynamically co-visualize time-series expression data with protein-protein interaction networks, here we examine all hubs with one or two interaction interfaces [10] and the interactions they facilitate during the yeast cell cycle. Since these hubs have many interaction partners, but a maximum of two interfaces, interaction between the hubs and their partners can only occur one or two at a time. Our analysis suggests the existence of two regulatory processes used by the cell to control the interactions of date hub proteins with their partners.

## Methods

### Data Sources

*S. cerevisiae* protein-protein interaction data was from Bertin *et al.* (2007) [19] and Kim *et al.* (2006) [10]. Bertin *et al.* is a metadataset that includes high-quality interaction data for 2,559 proteins linked by 5,996 interactions from large scale two-hybrid screens and immunoprecipitation studies. Interactions are present in this dataset if supported by multiple lines of evidence. The protein interaction data is thus considered high quality but is potentially prone to false negatives. Kim *et al.* similarly gathered interaction data from previous datasets; they filtered this data based on independent genomic features and Bayesian integration, and further filtered this to keep only the interactors which were seen to interact through Pfam domains (using iPfam). This resulted in a network of 873 proteins linked by 1,269 interactions, which can be also considered as a network of high-confidence interactions. Due to the Kim *et al.* dataset being focused on hubs and their interfaces, it was combined with Bertin *et al.* to assist in the construction of networks several steps beyond the hub proteins. Hubs and their classification as ‘singlish’ (one or two interaction interfaces) or ‘multi interface’ (three or more interaction interfaces) were from Kim *et al.* according to their structural analysis.

*S. cerevisiae* genome-wide time course expression data was obtained from Spellman *et al.* (1998) [20]. This contains expression values for each gene at multiple points throughout the cell cycle during normal growth. Data was used from the following experiments (i) α-factor arrest (6,153 genes, 18 sample points over 119 min), (ii) cdc15-2 arrest (5,983 genes, 24 sample points over 290 min), and (iii) Cdc28 arrest (6,150 genes, 17 sample points over 160 min). These data were smoothed using the loess function in R (span 0.5) [21] to assist in the continuous viewing of the time course data within GEOMI plugins, as in Goel *et al.* (2011) [16].

### Network Visualisation Software and Analysis

The GEOMI visualization platform [16], [22] was used for the four-dimensional analysis of protein-protein interaction networks, using real time rendering. We undertook network analysis for every singlish hub that was reported [10]. Time course data was co-visualized with localized networks, using the Four Dimensional Network Plugin [16]. Networks in GEOMI were seeded with the hub, and extended by 1 or 2 steps, as previously described [16]. Through visual and graphical analysis, hubs were classified as periodic (dynamic) or non-periodic (static); these classifications were further validated by reference to Cyclebase [23] and SCEPTRANS [24]. The expression of hub interaction partners were then analysed to determine similarities or differences between periodic and non-periodic hubs in terms of the nature of their interactions. A consistent expression threshold of −0.2 was used, below which proteins in the network and their interaction edges were not displayed. This threshold was set below the lower limit of expression of non-periodic hubs so as to minimize network disruption from stochastic gene expression. One exception, however, required a threshold of −0.1, due to its low amplitude of periodic expression. Hubs showing opposite, similar or sequential expression as compared to their interaction partners were identified. Finally, hubs were functionally classified according to their protein domains (from Pfam [25], SGD [26] and Uniprot [27]) and interaction models were built for hubs based on network visualizations and with documentation from the literature.

## Results

### Dynamic and Static Singlish Hubs

To determine if the cell has specific regulatory processes to control the interactions of date hub proteins with their partners, real time rendering [16] was used to examine all 36 singlish hubs in the yeast interactome during the cell cycle. Similarly to previous studies [15], [18], we defined proteins as dynamic or static depending on the periodicity of their expression within the cell cycle. We report 10 dynamic and 11 static hubs (Table1). Interestingly, we observed that 20 of these singlish hubs fell into two different and complementary classes; one where the hub is dynamic and at least some of the interaction partners are constitutively expressed, the other where the hub is static and some of its partners are dynamic. We also observed that the hubs within these groups were functionally related, so describe these hubs in related groupings, below. The remaining 15 hubs did not show any cell cycle-associated periodicity, and/or showed noisy or stochastic expression (Table 1). This is to be expected as not all hub proteins or their interaction partners will participate in cell cycle processes. Hubs and their partners that do not show cell-cycle regulated expression are not discussed any further here.

**Table 1:** Singlish hub proteins.

Hub Protein	Interaction Interfaces^a^	Domains^b^	Hub Classification^c^
**Bud Formation**			
Boi2	2	SH3_1,SAM_2,PH	Static
Cdc42	2	Ras	Static
Cla4	2	PH, PDB(CRIB), Pkinase	Dynamic
**Cyclin Proteins**			
Cdc28	2	Pkinase	Static
Clb1	2	Cyclin_N, Cyclin_C	Dynamic
Clb2	2	Cyclin_N, Cyclin_C	Dynamic
Clb3	2	Cyclin_N, Cyclin_C	Dynamic
Clb4	2	Cyclin_N, Cyclin_C	Dynamic
Clb5	2	Cyclin_N, Cyclin_C	Dynamic
Clb6	2	Cyclin_N, Cyclin_C	Dynamic
Cln1	2	Cyclin_N	Dynamic
Cln2	2	Cyclin_N	Dynamic
Cln3	2	Cyclin_N	Dynamic
Pho85	2	Pkinase	Static
Yta7	1	AAA, bromodomain	Static
**Structural**			
Act1	2	Actin	Static
Mlc1	2	−	Static
**Protein Sorting/Transport**			
Gdi1	1	GDI	Static
Mrs6	2	GDI	Static
Vps21	2	Ras	Static
Ykt6	2	Longin, Synaptobrevin	−
Ypt52	2	Ras	Static
**Others**			
Gsp1	2	Ras	−
Hsc82	2	HATPase_c, HSP90	−
Hsp82	2	HATpase_c, HSP90	−
Mtr3	1	RNase_PH	−
Ras2	2	Ras	−
Sgn1	1	RRM_1	−
Slt2	2	Pkinase	−
Snf1	2	Pkinase, UBA_2	−
**Ribosomal RNA Processing**			
Dbp8	1	DEAD, Helicase_c	−
Dbp9	1	DEAD, Helicase_c	−
Drs1	2	DEAD, Helicase_c	−
Has1	1	DEAD, Helicase_c, DUF4217	−
Hca4	1	DEAD, Helicase_c, DUF4217	−
Spb4	1	DEAD, Helicase_c, DUF4217	−

a Number of interactions interfaces as described by Kim *et al.* [10].

b Domains and abbreviations thereof from Pfam [25].

c Hub classified as ‘Dynamic’ if it shows periodic expression throughout the cell cycle. Hub classified as ‘Static’ if it has non-periodic expression throughout the cell cycle and it has at least one periodic interaction partner. Hubs with missing or low quality expression data not classified, and are shown with a hyphen.

### Dynamic Hubs - Bud Formation/cytokinesis

**Figure 1:**
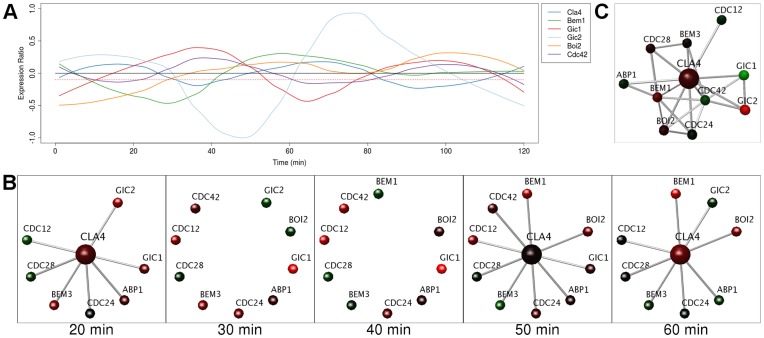
Cla4 is a dynamic hub with many static interaction partners. (**A**) Graph of expression ratios in the cell cycle, over time, showing periodic expression of Cla4 and its dynamic interaction partners. Red dotted line indicates threshold used in the network analysis. (**B**) Frames from the real-time rendering animation (threshold −0.1) from the network of Cla4 and its interaction partners. At each time point, nodes (representing proteins) map gene expression data from the cell cycle to a green/black/red color gradient. Proteins and their interactions are hidden when their expression at any point in time falls below the threshold. When Cla4 is present, many of its interaction partners are also expressed, suggesting that they will compete with each other to interact with Cla4 at those times. (**C**) Network of Cla4, its interaction partners, and their interactions with each other. This demonstrates the competition that Cla4, Gic1 and Gic2 may have in their interaction with Cdc42.

Cla4 kinase activity is required for septin ring proteins to localize and assemble the septin [28], [29]; this occurs after it binds to Bem1. Following this, it is activated by Cdc42 [29] which, in turn, brings about the phosphorylation of the other septin ring proteins [30], [31]. There are multiple lines of evidence to suggest that Cla4 has periodic expression; it is controlled in a cell cycle manner [32], [33], is involved in the dynamics of septin ring assembly [34] and documented as periodic in SCEPTRANS [24], [35]. However the expression peak amplitude of Cla4 was less than 0.2, which would not permit real time rendering using the −0.2 default threshold. A real time rendering threshold was thus set at −0.1 to reveal the periodic expression of Cla4 within the network. Cla4 was found to be a dynamic hub during the cell cycle, with peaks of expression at times 20 and 60 minutes (Figure 1A) which results in it disappearing from the interaction network at times 30–40 minutes, along with the non-hub proteins Bem1 and Gic2 (Figure 1B). This is distinct from five of its partners (Abp1, Bem3, Cdc12,24,28) which showed non-periodic or stochastic expression.

Cla4 contains a PBD domain (also known as CRIB); this is responsible for its interaction with, and activation by activated GTP-bound Cdc42 [29]. Our network analysis predicts that, during peak Cla4 expression, there will be competition between the septin ring proteins to access and be phosphorylated by the Cla4 protein kinase interface. This includes proteins Cdc12 and Cdc24. Non-septin proteins are not suggested to be phosphorylated by Cla4 (Abp1, Bem1,3, Boi2, Cdc28,42, Gic1,2) and as a result these are unlikely to compete for access to the Cla4 kinase as they also do not show substantial overlap of expression with Cla4. Interestingly, the non-septin proteins Gic1 and Gic2 contain PDB (CRIB) domains. When co-expressed with Cla4 these will all compete to interact with Cdc42 (e.g. times 50–60 minutes). These can be seen when all interactions in the network from Figure 1B are shown (Figure 1C). Bem1 is required to maintain Cdc42 at the site of polarized growth, and for the phosphorylation of Cdc24 by Cla4 [31]. Bem1 is visible in the network at similar times to that of Cla4, although its expression peaks and troughs precede Cla4 by ∼10 minutes (Figure 1A,B).

### Dynamic Hubs - Cln1-3 and Clb1-6

Cyclin-dependent kinases (CDKs) are protein kinases responsible for regulation of the cell cycle. CDKs by themselves have little to no activity, but interact with cyclin proteins to form an active cyclin-CDK complex [36]. There are 6 known CDKs in yeast: Cdc28, Pho85, Kin28, S2b10/Cdk8, Sdv1/Bur1, Ctk1 and there are 23 cyclins that provide the specificity of each cyclin-CDK complex. Each CDK may have many cyclins associated with it, and each cyclin-CDK complex may have many substrates. A number of cyclins and associated targets are known to be `singlish' hubs [10].

**Figure 2:**
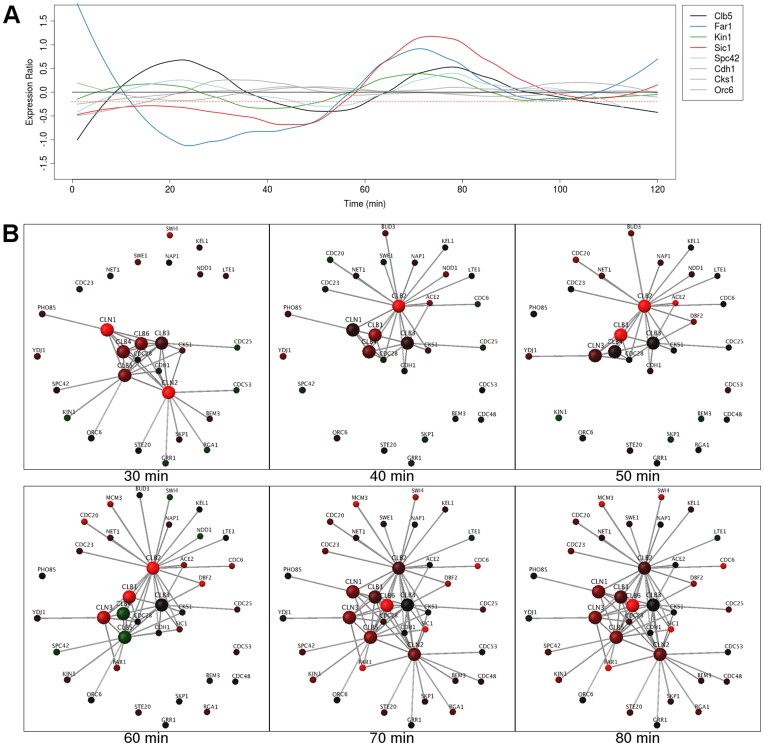
Cln1-3 and Clb1-6 are dynamic hubs with many static interaction partners. (**A**) Graph of expression ratios in the cell cycle, over time, showing periodic expression of cyclin Clb5 and its interaction partners. Dynamic interaction partners shown in color and static interaction partners are in gray. Red dotted line indicates threshold used in the network analysis. (**B**) Frames from the real-time rendering animation (threshold −0.2) from the network of the cyclins Cln1-3 and Clb1-6 and their interaction partners. At each time point, nodes (representing proteins) map gene expression data from the cell cycle to a green/black/red color gradient. Proteins and their interactions are hidden when their expression at any point in time falls below the threshold. When each cyclin is present, many of their interaction partners are as well, suggesting that they will compete with each other to interact with the cyclins.

Cyclins Cln1-3 are associated with Cdc28. In our network-based analysis, Cln1-3 showed dynamic expression during the cell cycle whereas their interaction partners were constitutively expressed. The peak expression order for the cyclins was 65 minutes (M/G1) for Cln2 and then 25/85 minutes (G1) for Cln1 and Cln2 [20]. The Cln3 and Cln1/2 hubs disappear from the network during their troughs of expression (times 30 and 50–60 minutes, respectively, Figure 2B) however most of the interaction partners of these proteins, excluding Cdc48, Far1 and Sic1, show constant expression. This suggests that there will be competition amongst the targets of the CDKs for the interaction interfaces of Cln1-3. This agrees with the well-described progression of the cyclins [36], [37].

The expression patterns of the cyclin hubs (Clb1-2 and Clb4-6) are dynamic with respect to their interaction partners. Similar to Cln1, Cln2 and Cln3, they interact with Cdc28 to form an active cyclin-CDK complex. At time 30 minutes, proteins Clb1 and Clb2 are downregulated and absent from the network, yet 11 of their interaction partners are still expressed (Figure 2A). Clb2 is strongly expressed at and between times 40 and 80 minutes (Figure 2B), whereupon its partners will compete to interact with it. In contrast to Clb2, Clb4 is downregulated at times 60 to 80 minutes but remains visible in the network. The hub proteins Clb5 and Clb6 are absent from the network at times 40–50 and at 40–60 minutes respectively, but strongly expressed at other times (Figure 2B). Figure 2A shows Clb5, and its interaction partners, as an example of the differences in expression between cyclins and their targets. During their downregulation, 4 out their 6 interaction partners are not downregulated. In this manner, proteins Clb1, Clb2 and Clb4 to Clb6 are observed to be dynamic hubs with expression different to the majority of their interaction partners. In these cases, there is likely to be competition between the Clb proteins and their partners when the Clb proteins are maximally expressed.

### Static Hubs - Cdc28 and Pho85

**Figure 3:**
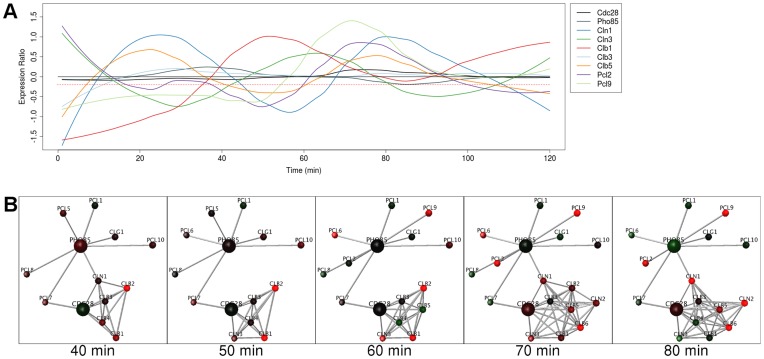
Cdc28 and Pho85 are static hubs with many dynamic interaction partners. (**A**) Graph of expression ratios in the cell cycle, over time, showing non-periodic expression of the CDKs Cdc28 and Pho85 and the dynamic cyclins they interact with. Red dotted line indicates threshold used in the network analysis. Cyclins that act redundantly with other cyclins have not been shown (Cln2, Clb2,4,6). (**B**) Frames from the real-time rendering animation (threshold −0.2) from the network of Cdc28 and Pho85 and their interacting cyclins. At each time point, nodes (representing proteins) map gene expression data from the cell cycle to a green/black/red color gradient. Proteins and their interactions are hidden when their expression at any point in time falls below the threshold. The CDKs are present throughout the cell cycle, but their cyclins are not. Thus the cyclins do not have to compete with all the other cyclins to interact with a CDK but only those expressed at similar times.

Cdc28 and Pho85 are the catalytic subunits of CDKs (cyclin-dependent kinases). Pho85 is homologous to Cdc28 and is suggested to have similar or overlapping roles in the cell cycle [38]. Cdc28 and Pho85 can only interact with one cyclin at a time, so their interactions are mutually exclusive. Their cyclins are: Cln1-3 and Clb1-6 for Cdc28 [39] and Pcl1,2, Pcl5-10, Pho90 and Clg1 for Pho85 [40]. All of these except for Pcl5 are represented in our interaction datasets. These can be observed in Figure 3, where the proteins Cdc28 and Pho85 have constitutive expression and dynamically expressed interaction partners. The cyclins are not visible in the network during their troughs of expression within the cell cycle. Cdc28 and Pho85 can therefore be classified as static hubs. For Cdc28 the cyclin progression is well documented [39], [41]; Cln1-3 are upregulated at G1, Clb5,6 at S and Clb1-4 at G2. This matches our observations well, with the exception of Clb3, whereby Cln1,2 are upregulated at 70–80 min, Cln3 at 60 minutes, Clb5,6 at 70–80 minutes, Clb4 at 40 minutes and Clb1,2 at 40–60 minutes. For Pho85, only 4 of its cyclins are known to be cell cycle associated; these are Pcl1,2,7,9 [38]. Two of these are seen as dynamic within the network; Pcl2 and Pcl9 are upregulated and visible (Figure 3B) at times 60–80 minutes (G1). It is proposed that CDKs are examples of constitutively expressed hubs with dynamic partners, where the substrate specificity of the kinase interface is controlled by the abundance (or regulation) of their interacting cyclins. This is consistent with previous reports that specificity of kinases can be changed by their protein interaction partners [42], [43].

### Static Hubs - Structural Proteins

**Figure 4:**
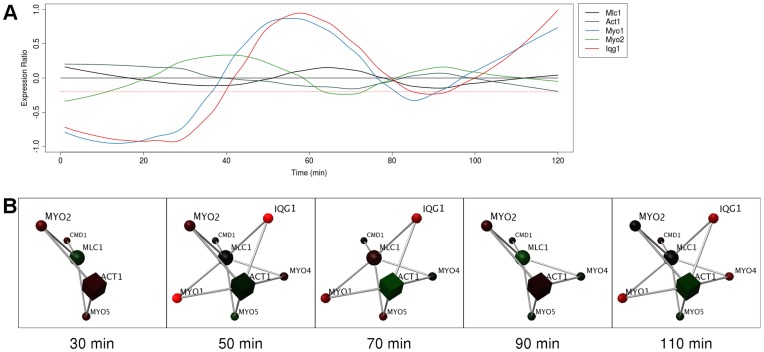
Mlc1 and Act1 are static hubs with some dynamic partners. (**A**) Graph of expression ratios in the cell cycle, over time, showing non-periodic expression of Act1 and Mlc1 and their interaction partners. Red dotted line indicates threshold used in the network analysis. (**B**) Frames from the real-time rendering animation (threshold −0.2) from the network of Mlc1 and Act1 and their interaction partners. At each time point, nodes (representing proteins) map gene expression data from the cell cycle to a green/black/red color gradient. Proteins and their interactions are hidden when their expression at any point in time falls below the threshold. Act1 and Mlc1 are present throughout the animation, but some of their interaction partners are not. When interaction partners are present, they are suggested to not compete with each other to interact with Act1 and/or Mlc1 due to their staggered expression peaks.

Myosin light chain 1 (Mlc1) and actin 1 (Act1) are hub proteins with two interaction interfaces [10]. While both proteins are known to be structural due to the role they play as cytoskeletal elements, they interact with a number of proteins to change the shape of the cell during mitosis, bud growth, actin organization and endocytosis [44]. We previously noted that the expression of Mlc1 shows little change in the cell cycle, yet its interaction partners show strong expression peaks at mutually exclusive times during the cell cycle [16]. Here we report that Act1, an interaction partner of Mlc1, also shows little expression change throughout the cell cycle but that some of its interaction partners show strong periodic expression (Figure 4A). Its partners Iqg1 and Myo1 are downregulated at time 30 and 90 minutes (these times are one cell cycle apart) whereas another partner Myo2 is downregulated at time 70 minutes (Figure 4B). Importantly, as the expression peaks of these partners are staggered, this will reduce competition for interaction with Act1 at certain times in the cell cycle. It has been shown elsewhere that Act1, Myo1 and Iqg1 all accumulate in the neck ring late in anaphase [45] (∼70 minutes); this is reflected in our network model.

### Static Hubs - Protein Transport/Sorting

**Figure 5:**
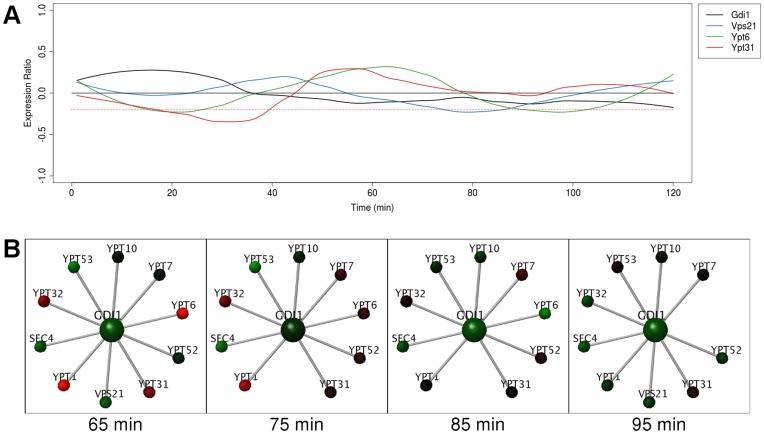
Gdi1 is a static hub with a few dynamic interaction partners. (**A**) Graph of expression ratios in the cell cycle, over time, showing non-periodic expression of Gdi1 and its interaction partners. Red dotted line indicates threshold used in the network analysis. (**B**) Frames from the real-time rendering animation (threshold −0.2) from the network of Gdi1 and its interaction partners. At each time point, nodes (representing proteins) map gene expression data from the cell cycle to a green/black/red color gradient. Proteins and their interactions are hidden when their expression at any point in time falls below the threshold. The dynamic interaction partners of Gdi1 will compete with other proteins only when expressed at the same time.

Gdi1 is a GDP dissociation inhibitor, it is involved in the recycling of Sec4/Ypt/rab family proteins from their target membranes back to the vesicular pool [46]. In a previous study [16] we reported that Ypt52 showed constitutive expression and, with its 4 dynamic partners, formed a static hub. Here, we report that Gdi1 is similarly regulated. Gdi1 has 10 interaction partners, 3 of which are dynamically expressed in the cell cycle, albeit stochastically (Figure 5A). These proteins are Vps21 (downregulated and hidden from the network at 75–85 minutes), Ypt6 (hidden at 85–95 minutes) and Ypt31 (hidden at 20–40 minutes, not shown); they will compete to interact with Gdi1 if spatially co-located. Gdi1 thus, in part, fits the model of a static hub with dynamic interaction partners. However, there are 7 remaining interaction partners that are non-periodically expressed (Sec4, Ypt1,7,10,32,52,53, Figure 5B). These partners reflect the important role of Gdi1 in vesicle trafficking; Gdi1 depletion causes blocks in protein transport at multiple stages of the secretory pathway [46]. The many interactions of Gdi1 are thus consistent with a previously noted feature of date hub proteins, which show interactions with different proteins in different parts of the cell and/or at different times [1].

### Other Hubs

During cell synchronisation, it is expected that proteins will either show periodic expression (at least one peak and trough of expression during a cell cycle), constitutive expression (zero or little fold change) or stochastic but non-periodic changes (which includes noise). There were 15 singlish hub proteins which were not possible to classify as static or dynamic. These included the trafficking protein Ykt6, a number of ribosomal RNA processing proteins and 8 others (Table 1). These hubs had noisy expression data or showed expression patterns that were inconsistent between the two cell cycles and/or the three different conditions that were used to synchronize cells (alpha factor, CDC28 and CDC15 temperature sensitive mutants). When visualized in networks with real-time rendering, they did not show any consistent patterns, despite some of these being proposed to have periodic expression [23].

The singlish hub proteins Hca4, Has1, Spb1, Dbp8, Drs1, Dbp9 and Spb4 are all involved in rRNA processing and ribosome biogenesis. They are known to be of periodic expression, associated with the generation of new ribosomes in preparation for mitosis [47]. With the exception of Spb1, they interact directly with each other in networks. In Spellman *et al.* [20] it was shown that these proteins had an initial and co-ordinated downregulation of expression, which was not seen during the later cell cycles. In real-time rendering of the network, due to the co-ordinate expression of the proteins, all nodes appear or disappear at the same time (data not shown). This type of regulation is more commonly seen for multiprotein complexes that involve proteins with large numbers of interaction interfaces [1] and is seen for a large number of interactions in the yeast cell [48]. It highlights that not all singlish interface proteins in the cell show differences in expression patterns compared to their partners.

## Discussion

This study has used 4-D real time rendering of networks [16] to explore the regulatory relationships of hub proteins, and their interaction partners, in the yeast cell cycle. In particular, we sought to understand whether hub proteins that have one or two interaction interfaces, or their many interaction partners, are subject to restrictions on expression to reduce competition at interaction interfaces. Interestingly, this analysis revealed that 20 singlish hub proteins (out of a total of 35 in the yeast proteome) showed cell cycle-associated expression changes which involved either the hub showing static expression along with periodic expression of partners, or the hub showing periodic expression but its partners being constitutive.

Dynamic hubs with static partners can be considered as ‘competitive hubs’. Their interaction partners will compete for the interaction interface of the hub and the success of any interaction will be dictated by the kinetics of interaction (abundance and affinity) and subcellular localisation. Dynamic hubs were found to predominantly be cyclins. By contrast, static hubs with dynamic partners are ‘non-competitive hubs’. Regulatory mechanisms are finely tuned to lessen the presence and/or effects of competition between the interaction partners of the hub. In this case, the regulation of a protein-protein interaction is likely to rely on dynamic control of protein expression and regulation of half-life of the proteins involved, rather than the interaction affinity. This is consistent with recent observations that interaction specificity of proteins cannot always be explained by differences in affinities but may also involve the regulation of expression of proteins that compete for an interaction partner [49]. In contrast to dynamic hubs, static hubs with dynamic partners were functionally associated with the cytoskeleton and cellular trafficking. It should be noted that, due to the uncertainty of interaction interfaces (and what proteins interact through which interface), the interaction partners of dynamic or static hubs might act in cooperation/coordination rather than in any competitive manner. For example, the phosphorylation of a hub by interaction with a kinase may be required for it to interact (even at the same interface) with a second protein; in this case the kinase and second protein are not in strict competition.

It has been previously suggested that the majority of proteins that interact within the cell are co-expressed. Indeed this has previously been used to filter interaction datasets for false positives [1], [48], [50], [51]. However, we have shown that some singlish hubs and their interaction partners have complicated patterns of non co-expression. There are a number of examples whereby the cell uses sophisticated mechanisms to control how and when proteins interact. One of these is the just in time assembly of protein complexes. In this, the majority of proteins in a complex show constitutive expression during the cell cycle however the complex is not functional until the final subunit is expressed at a certain time [15]. It has also been reported that some hubs show high correlation of expression with their partners only at specific times of the cell cycle, suggesting the presence of phase-specific networks [18]. It will be fascinating to examine if other singlish hubs and their interaction partners also show expression patterns of dynamic hubs/static partner or static hubs/dynamic partners in circumstances other than in the cell cycle. The use of protein-fragment complementation assays is likely to be useful for the *in vivo* validation of these effects [52], as opposed to traditional two-hybrid systems.

It is important to acknowledge that the temporal networks constructed and analysed in this study do not consider several factors. Many of these issues were discussed in detail previously [16] and include limitations in the quality of some time series gene expression data, the fact that gene expression is an imperfect proxy for protein abundance, a lack of knowledge and no consideration of the modification status of proteins, no consideration of protein abundance or half-life, no information available for protein affinity or dissociation constants, and false positives and negatives in the protein interaction datasets. It should also be noted that the real-time rendering of networks is sensitive to the levels of thresholds that are used to show or hide proteins. The network analysis conducted here relied on manual visual analysis, with a consistent threshold when possible. However analysis could also be done through the use of automated threshold determination, and network construction, or non-visual algorithmic and/or statistical analysis. In sum, real-time rendering provides a unique means to integrate time-series gene or protein expression data with protein interaction networks and thus provide insights into intracellular regulatory processes.
